# 肺癌免疫检查点抑制剂相关肺炎的危险因素、分子机制及多模态预警策略

**DOI:** 10.3779/j.issn.1009-3419.2025.101.19

**Published:** 2025-11-20

**Authors:** ZHAN Jurong, CHEN Xiudi, LI Na

**Affiliations:** 400010 重庆，重庆医科大学附属第二医院呼吸与危重症医学科; Department of Respiratory and Critical Care Medicine, The Second Affiliated Hospital of Chongqing Medical University, Chongqing 400010, China

**Keywords:** 肺肿瘤, 免疫检查点抑制剂相关肺炎, 危险因素, 分子机制, 早期预警机制, Lung neoplasms, Immune checkpoint inhibitor-associated pneumonitis, Risk factors, Molecular mechanism, Early warning mechanism

## Abstract

近年来，免疫检查点抑制剂（immune checkpoint inhibitors, ICIs）作为肿瘤治疗领域的革命性治疗手段，已在多种恶性肿瘤的临床实践中展现出显著疗效。随着临床应用的普及，其相关毒性反应已成为肿瘤免疫治疗领域亟待解决的关键问题。ICIs相关肺炎（ICIs-associated pneumonitis, CIP）特指ICIs治疗引发的肺部免疫相关不良事件（immune-related adverse events, irAEs），其发病机制目前尚未完全阐明。作为ICIs治疗罕见且严重的并发症之一，CIP具有起病隐匿、进展迅猛、预后不良及致死率高等临床特征，其临床表现与影像学征象呈现高度异质性。由于缺乏特异性生物标志物及客观检测指标，CIP的早期识别与诊断面临重大临床挑战。本文通过回顾既往文献及研究，总结了CIP在肺癌免疫治疗患者中的临床表现、危险因素、可能的分子机制、生物标志物及早期预警机制等的研究进展，旨在为CIP临床诊疗提供参考，为建立CIP早期筛查与精准诊疗体系提供理论依据。

## Abstract (en)

In recent years, immune checkpoint inhibitors (ICIs), as a revolutionary therapeutic approach in oncology, have demonstrated remarkable clinical efficacy across various malignant tumors. With the widespread clinical application of ICIs, their associated toxicities have emerged as a critical issue that urgently requires resolution in the field of cancer immunotherapy. ICIs-associated pneumonitis (CIP) specifically refers to immune-related adverse events (irAEs) of the lung induced by ICIs therapy, the underlying pathogenesis of which remains incompletely elucidated. As a rare yet severe complication of ICIs treatment, and CIP is characterized by insidious onset, rapid progression, poor prognosis, and high mortality rate, with highly heterogeneous clinical manifestations and radiological features. Due to the lack of specific biomarkers and objective diagnostic indicators, the early identification and diagnosis of CIP present significant clinical challenges. By reviewing previous literature and studies, this paper summarizes recent advances in understanding the clinical manifestations, risk factors, potential molecular mechanisms, biomarkers, and early warning systems of CIP in patients receiving immunotherapy for lung cancer. The aim of this article is to provide a reference for the clinical management of CIP and offer a theoretical basis for establishing an early screening and precision diagnosis and treatment system for this condition.

肺癌是起源于支气管黏膜或者腺体的肺部恶性肿瘤，其病变主要累及支气管、终末细支气管与肺泡。作为肺部原发性恶性肿瘤中最常见的类型，肺癌具有高度侵袭性及致命性，其发病率与致死率均居全球恶性肿瘤首位^[1]^。免疫检查点抑制剂（immune checkpoint inhibitors, ICIs）已成为多种实体肿瘤治疗的重要手段，在肺癌、尿路上皮癌、黑色素瘤等恶性肿瘤的临床实践中展现出明确的临床获益。然而，ICIs治疗引发的免疫相关不良事件（immune-related adverse events, irAEs）日益受到临床重视^[2]^。ICIs相关肺炎（ICIs-associated pneumonitis, CIP）是指由ICIs治疗引起的常见irAEs）之一。CIP作为具有较高临床危害性的irAEs类型，其3-4级重症病例如未能及时采取有效干预措施，可导致ICIs的短暂甚至长期停药，不仅显著制约抗肿瘤免疫治疗的连续性，而且存在进展为呼吸衰竭乃至死亡的风险。据研究^[3-5]^报道，相较于其他癌种，肺癌接受免疫治疗后并发CIP的风险具有显著临床差异，尤以鳞状细胞癌亚型为著。临床上若能早期识别及诊断CIP，部分预后良好患者仍具备进行免疫治疗再挑战的可行性^[6]^。

多项研究^[7-9]^报道CIP发生率为2.6%-33%。CIP作为ICIs治疗过程中潜在的严重临床并发症，危险因素的早期识别极为重要。由于CIP临床表现及影像学征象的显著异质性，且缺乏特异性生物标志物，故CIP诊断十分困难，目前主要依赖于临床表现和胸部高分辨率计算机断层扫描（high resolution computed tomography, HRCT）。CIP的发病机制与其发生发展及严重程度相关的危险因素目前尚不清楚^[10]^。临床CIP重症及急性加重患者死亡率高，目前亟需通过CIP相关危险因素及预警信号来早期区分和识别疾病的进展状态与病变程度。

## 1 CIP的概述

ICIs的抗肿瘤作用是通过激活自身免疫系统杀伤肿瘤细胞完成，目前临床上常用的ICIs主要包括程序性细胞死亡受体-1（programmed cell death protein-1, PD-1）及其配体1（programmed cell death ligand 1, PD-L1）、抗细胞毒T淋巴细胞相关抗原4（cytotoxic T-lymphocyte antigen 4, CTLA-4）^[2]^。CIP是一种由ICIs治疗引起的潜在的临床、影像学和病理学表现各异的致命性irAEs。Wang等^[11]^报道CIP是抗PD-1/PD-L1治疗中最常见的致命原因，占相关死亡原因的35%。CIP是ICIs治疗期间及治疗后出现与胸部CT上新出现的浸润影有关的呼吸道症状和体征表现，其组织病理学特征包括不同程度的炎症细胞浸润、肺泡损伤和纤维化。由于CIP的临床表现和影像学特征缺乏特异性，且癌症治疗相关间质性肺疾病（cancer treatment related interstitial disease, CT-ILD）是一种排他性的诊断，故需要排除本身肺癌进展、经痰和（或）支气管肺泡灌洗（bronchoalvelar lavage fluid, BALF）等病原学检测验证的新发感染^[12]^，及与弥漫性肺泡出血、心功能不全所致的心源性肺水肿等进行鉴别。

## 2 CIP的临床表现

CIP的发作时间存在显著个体差异，可自治疗启动后数小时至数年不等。多数病例呈隐匿性起病且临床表现多样，从无症状的影像学改变到缓慢进展或急性加重等多种表现形式。患者可能出现的症状包括但不限于咳嗽、咳痰、发热、疲劳、胸痛或咯血、呼吸困难乃至发展至重症肺炎、呼吸衰竭^[2,6,13]^。肺部听诊可能显示正常的呼吸音或典型的“Velcro啰音”，疾病晚期可能伴有右心室功能障碍和肺动脉高压的体征，其症状与其他并发症状相似。

CIP的典型影像学表现可分为4种主要类型：机化性肺炎（organizing pneumonia, OP）、过敏性肺炎（hypersensitivity pneumonitis, HP）、弥漫性肺泡损伤（diffuse alveolar damage, DAD）及非特异性间质性肺炎（nonspecific interstitial pneumonia, NSIP）^[14,15]^，其中NSIP占绝大多数，而OP、DAD虽然罕见但病情严重。目前主要推荐HRCT用于CIP的早期识别和诊断。CIP组织学表现多样且缺乏相对特异性，因此无法仅通过组织学确诊CIP^[16,17]^。BALF在CIP中的诊断敏感性有限，经支气管肺活检及外科肺活检作为侵入性检查且合并众多活检相关风险，故临床不作为常规推荐。

## 3 CIP的分子机制

ICIs的出现彻底改变了肿瘤的治疗格局，但目前其分子机制尚未完全阐明。CIP的发生涉及复杂的免疫系统调节失调，核心在于免疫检查点通路抑制被解除后导致的T细胞过度激活和免疫失衡，以及下游炎症信号通路的级联放大效应。

### 3.1 基础免疫失衡，细胞免疫过度活化

#### 3.1.1 细胞过度活化

ICIs通过阻断相应信号通路，解除对T细胞的抑制，攻击肿瘤细胞的同时亦可能错误地识别和攻击正常肺组织。T细胞异常过度激活是CIP的直接驱动因素，可引发下游炎症反应。多项实验对CIP患者肺组织和BALF中淋巴细胞进行检测，发现CD4^+^ T淋巴细胞和CD8^+^ T淋巴细胞富集^[4,18]^，并出现CD4^+^/CD8^+^比例倒置，均反映了淋巴细胞介导的超免疫反应^[19,20]^。一项关于T细胞受体测序的研究^[18]^发现，CIP患者病变肺组织中T细胞与肿瘤浸润淋巴细胞在T细胞受体表达谱上存在显著重叠。ICIs治疗在激活细胞免疫功能的同时，也可导致T细胞亚群失衡（如Th1/Th17极化）。

#### 3.1.2 中性粒细胞-免疫轴失衡

中性粒细胞浸润和激活在CIP中可能导致免疫轴失衡。中性粒细胞-免疫轴通过促进炎症细胞浸润、放大T细胞介导的自身免疫及驱动肺纤维化进展，在CIP发病中起着关键作用。基于外周血中性粒细胞与淋巴细胞比值（neutrophil and lymphocyte ratio, NLR）和乳酸脱氢酶（lactate dehydrogenase, LDH）水平构建的肺免疫预后指数（lung immune prognostic index, LIPI）与ICIs治疗患者的预后和生存结局密切相关^[21]^。高NLR可能提示全身性炎症状态，有助于CIP高危患者的风险分层。中性粒细胞浸润可释放活性氧（reactive oxygen species, ROS）、蛋白酶（如弹性蛋白酶）及促炎因子，如白细胞介素（interleukin, IL）-8、肿瘤坏死因子-α（tumor necrosis factor-α, TNF-α），直接损伤肺组织并放大炎症级联反应和形成细胞因子风暴^[22]^。研究^[23,24]^发现CIP的出现与包括C反应蛋白（C-reactive protein, CRP）、IL-6、IL-10、IL-17A、IL-35、干扰素-γ（interferon-γ, IFN-γ）等在内的各种炎性细胞因子和趋化因子之间存在关联。

#### 3.1.3 预先存在的自身抗体的激活

自身抗体是指由人体免疫系统产生的、专门针对自身正常组织、细胞、细胞成分或分泌物质的异常抗体，其在CIP中发挥着重要作用。Miura等^[25]^通过直接检测患者血浆中的抗CD74自身抗体水平，观察到在≥2级irAEs患者中基线CD74自身抗体水平显著升高。Tahir等^[26]^通过液体活检技术测定接受ICIs治疗患者血浆的自身抗体水平，也发现CIP患者血浆抗CD74自身抗体水平较基线水平上升1.75倍，且血浆中高抗CD74自身抗体水平者更易发生CIP。

#### 3.1.4 交叉抗原反应

肿瘤细胞表面存在与正常肺组织形态相似的抗原。Zhang等^[27]^发现CIP患者的BALF中T细胞明显富集，在CD4^+ ^T细胞亚群中，滤泡辅助性T细胞（Tfh）样T细胞高度富集并表现出与炎症和克隆扩增相关的特征，提示存在抗原驱动的特异性免疫应答，这为交叉抗原反应提供了直接证据。

### 3.2 细胞因子风暴及关键信号通路异常激活（图1）

**图 1:**
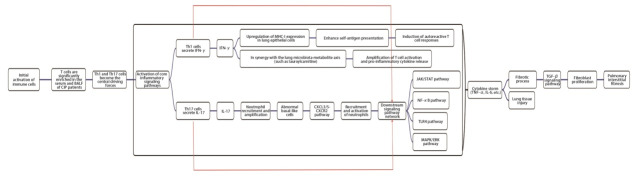
CIP发病机制

细胞因子水平的升高在急慢性炎症中均可引发剧烈的炎症反应与组织损伤，异常激活的T细胞可产生过量的细胞因子引发细胞因子风暴，打破免疫平衡，从而促进CIP的发生和发展^[28]^。单细胞分析显示CIP患者BALF中T细胞显著富集，其中Th1（分泌IFN-γ）和Th17（分泌IL-17）细胞驱动的炎症信号通路是CIP的核心机制^[18]^。Zheng等^[29]^将重度CIP患者的BALF进行单细胞测序，发现了一群富集的异常基底样细胞，分析其可能通过CXC趋化因子配体3/5（chemokine CXC ligand 3/5, CXCL3/5）/CXC趋化因子受体2（C-X-C motif chemokine receptor 2, CXCR2）通路上调SRY框转录因子9（SRY-box 9, SOX9），以募集和激活中性粒细胞，进而激活免疫系统从而导致重度CIP。活化的Th1/Th17细胞通过分泌IFN-γ、IL-17等促炎因子，激活肺组织内Janus激酶/信号转导和转录激活（Janus kinase/signal transducer and activator of transcription, JAK/STAT）、核因子-κB（nuclear factor-κB, NF-κB）、Toll样受体4（Toll-like receptor 4, TLR4）和丝裂原活化蛋白激酶/细胞外信号调控激酶（mitogen-activated protein kinase/extracellular signal-regulated kinase, MAPK/ERK）等信号通路，传递炎症信号并驱动炎症级联反应，形成局部细胞因子风暴，导致免疫稳态失衡并加重肺组织损伤^[30]^。IFN-γ通路的失调具有双重病理作用：一方面，通过上调肺上皮细胞组织相容性复合体I类（major histocompatibility complex class I, MHC-I）表达，增强自身抗原提呈能力，诱发自身反应性T细胞应答；另一方面，肺部菌群-代谢物轴（如月桂酰肉碱积累）可协同IFN-γ通路，进一步放大T细胞活化及促炎因子释放，形成恶性循环^[6,31]^。此外，转化生长因子-β（transforming growth factor-β, TGF-β）介导的成纤维细胞增殖在部分CIP患者中促进肺间质纤维化进展，与特发性肺纤维化存在部分重叠机制，提示纤维化亚型需差异化干预^[31]^。

CIP的分子机制研究为识别其临床危险因素提供了理论基础。在明确这些机制驱动的免疫应答失衡之后，将进一步分析其危险因素。

## 4 CIP的危险因素

CIP高危人群的危险因素筛查和预警目前尚缺乏权威、统一的标准。CIP预测生物标志物和风险分层工具不足，急性加重和重症CIP管理策略缺乏证据，综合目前的最新研究进展，其潜在危险因素可简要概括为患者自身因素、治疗相关因素、炎症生物标志物三个方面。

### 4.1 患者自身因素

#### 4.1.1 人口学特征

年龄、性别、身体质量指数（body mass index, BMI）老年患者因免疫力下降，更易受到ICIs“非靶向”效应的影响，>70岁的老年患者罹患CIP的风险更高^[2]^。亚洲患者发生率显著高于非亚洲患者，种族差异、遗传背景、环境因素均可能在CIP发病中起一定作用。另外肥胖也是CIP的重要风险因素，BMI升高、纤维蛋白原（fibrinogen, FIB）水平上升及肺弥散功能下降构成CIP的独立预测三联征，三者联合预测CIP的曲线下面积达0.806^[31]^。

#### 4.1.2 吸烟史及肺部基础合并症

吸烟者比非吸烟者CIP发生率更高，吸烟史是CIP的独立预测因子及危险因素^[32]^。既往存在肺部基础合并症，如间质性肺异常（interstitial lung abnormalities, ILAs）^[33]^，尤其是非胸膜肺弹力纤维增生症（non-pleuroparenchymal fibroelastosis, non-PPFE）^[34]^、慢性阻塞性肺疾病（chronic obstructive pulmonary disease, COPD）^[35]^、肺气肿、哮喘、气胸、胸腔积液、肺纤维化、肺部近期手术史等显著增加CIP风险；有研究^[36,37]^指出COPD是CIP发生的独立危险因素（OR=7.194）。

#### 4.1.3 肺功能状况

基线第一秒用力呼气容积/用力肺活量（forced expratory volume in one second/forced vital cappacity, FEV_1_/FVC）、一氧化碳弥散量（diffusion lung carbon monoxide, DLCO/pre%）降低及限制性通气功能障碍与CIP有关，DLCO降低是区分轻重症CIP的关键指标，与重症CIP风险存在显著负相关（P<0.001）^[38]^。

#### 4.1.4 肿瘤生物学特征

CIP的发生与原发肿瘤类型也密切关联。与黑色素瘤、肾癌等相比，肺癌患者的CIP患病率更高^[39]^，发病时间更短，尤其是非小细胞肺癌（non-small cell lung cancer, NSCLC），且鳞癌发生CIP的概率较腺癌更高^[8]^。

### 4.2 治疗相关因素

#### 4.2.1 ICIs类型

CIP的发生率随ICIs类别不同而有所区别。抗CTLA-4单药治疗的CIP发生率通常低于抗PD-1/PD-L1单药治疗，相较于PD-L1单抗，使用PD-1单抗的患者出现CIP的几率更高^[21,40]^。

#### 4.2.2 联合放化疗/靶向治疗

CIP在与PD-1/PD-L1抑制剂相关的致死性不良事件中占主导地位，ICIs联合疗法（如PD-1/PD-L1+CTLA-4）风险显著高于单药治疗，在联合治疗中发生率高达7.0%-10.0%^[41]^。

#### 4.2.3 序贯治疗

在ICIs治疗期间序贯使用表皮生长因子受体-酪氨酸激酶抑制剂（epidermal growth factor receptor-tyrosine kinase inhibitors, EGFR-TKIs）显著增加CIP的风险（OR=1.79, 95%CI: 1.34-2.38），形成“二次打击”效应，且序贯治疗组3-5级重度ILD风险及住院死亡率明显升高^[42]^。

#### 4.2.4 既往放疗史

既往放疗史会增加发生CIP的风险^[43]^。在抗肿瘤作用中放疗与免疫疗法存在协同作用，Noda-Narita团队^[44]^通过研究指出CIP优先发生于既往接受过放疗的放射区域内，但该研究并未明确说明特定放疗参数（特定时间、剂量、疗程等）与CIP的发生明确相关。

### 4.3 炎症生物标志物

#### 4.3.1 FIB

单变量和多变量分析均表明，高FIB水平是CIP发生率和死亡率升高的危险因素，是预测CIP三联征之一^[31]^。

#### 4.3.2 嗜酸性粒细胞绝对数（absolute eosinophil count, AEC）

CIP患者的AEC水平显著高于非CIP患者，AEC水平高的患者具有更高的客观缓解率和更长的无进展生存期（progression-free survival, PFS），且在接受ICIs治疗的患者中，AEC水平的基线特征与CIP风险和临床结局相关^[32,45]^。

#### 4.3.3 全身免疫炎症指数（systemic immune-inflammation index, SII）

高SII值通常反映更强的炎症负荷，与接受ICIs治疗的癌症患者的不良预后显著关联^[46]^。SII、NLR、全身炎症反应指数（systemic inflammatory response index, SIRI）、乳酸脱氢酶（lactate dehydrogenase, LDH）、CRP、白蛋白（albumin, ALB）水平等均与较差的总生存期（overall survival, OS）和PFS显著关联（P<0.001）^[47,48]^。

#### 4.3.4 细胞因子

CIP患者BALF中的CXCL13、TNF-α、IFN-α、IFN-γ、IL-6、IL-17A、IL-35等促炎因子水平显著升高^[5]^，提示CIP过度活跃的免疫反应和细胞因子风暴级联反应。

## 5 CIP的生物标志物

目前尚无CIP的特征性生物标志物，现阶段的研究主要包含血清和BALF中相关生物标志物发掘。研究^[5,32]^表明，NLR、血小板与淋巴细胞比值（platelet-to-lymphocyte ratio, PLR）和LDH的升高以及血红蛋白（hemoglobin, Hb）和ALB的降低均与CIP的发生有关。另外，一些新型生物标志物如涎液化糖链抗原-6（Krebs von den lungen-6, KL-6）、肺泡表面蛋白-D（surfactant associated protein D, SP-D）等肺特异性蛋白均显示出潜在的预警价值^[4]^。

### 5.1 KL-6

KL-6是诊断间质性肺炎的重要指标，主要反映并参与II型肺泡上皮细胞再生和损伤修复过程^[49]^，其动态变化是疾病进展和预后的“风向标”。有研究^[50]^显示，KL-6水平升高（>500 U/mL）与CIP的严重程度和预后不良显著关联，其早期检测有助于及时干预CIP，预防治疗中断并减少免疫抑制剂的用量。

### 5.2 细胞因子和高迁移率族蛋白B1（high-mobility group box 1 protein, HMGB1）

血清及BALF中的细胞因子和HMGB1可作为CIP的潜在生物标志物。Kikuchi等^[51]^发现HMGB1高水平组的CIP发病率明显高于HMGB1低水平亚组；IL-8在CIP发生时显著升高（临界值≥35.655 pg/mL），与严重CIP及其他irAEs有关；IL-17A在基线水平高可独立预测早发型CIP；高水平IL-10是重症CIP的危险因素（P<0.05）。

### 5.3 BALF中细胞因子谱和细胞亚群特征

CIP患者BALF有其独特的细胞因子谱，Tfh样T细胞、过度激活的CD8^+ ^T细胞和效应记忆T细胞在CIP患者的BALF中富集，树突状细胞抗原呈递能力升高^[44]^。CIP患者BALF中的促炎细胞因子显著升高，包括TNF-α、IFN-α、IFN-γ等^[23,24]^。

### 5.4 BALF中淋巴细胞

BALF中淋巴细胞增多可作为某些临床病例中诊断CIP的辅助手段，其BALF表现为以淋巴细胞增多及CD8^+^ T细胞优势为特征的免疫表型。黄慧教授团队^[52]^通过一项单中心回顾性研究指出，CIP患者普遍存在淋巴细胞优势型肺泡炎（81%, 34/42），其BALF淋巴细胞比例显著高于健康人群及肺部疾病对照组（P<0.01），且与常见不良事件通用术语标准（Common Terminology Criteria for Adverse Events, CTCAEs）分级无关（3-5 vs 1-2级，*P*=0.58）。

## 6 CIP的预测因素及风险预测模型

### 6.1 ILAs ILAs是CIP的危险因素^[53]^。

#### 6.1.1 胸膜下纤维化ILAs（subpleural fibrotic ILAs, SF-ILAs）

SF-ILAs是NSCLC患者CIP发展的独立强预测因子。开始ICIs治疗前存在SF-ILA与CIP风险增加有关，该类患者出现放射学ILAs进展的比例约为非SF-ILAs患者的2.5倍^[53]^。SF-ILAs患者的PFS和OS也比非SF-ILAs患者短，且任何级别及≥3级CIP的发病率也更高。

#### 6.1.2 非纤维化ILAs

非纤维化ILAs的存在是接受ICIs单药治疗的NSCLC患者早发性CIP的显著风险，Isobe等^[54]^提出保持全肺磨玻璃影（ground-glass opacity, GGO）百分比、年龄和病理类型这3个预测因子不变的预测模型显示出更好的判别能力和更准确的预测能力。Qi等^[4]^揭示了预先存在的非纤维化ILAs是导致ICIs单药治疗引起的早期CIP的独立风险因素。

### 6.2 肠道微生物菌群

肠道微生物及其代谢产物在调节宿主免疫反应中发挥着重要作用^[55]^。在CIP患者中，通过肠-肺轴影响肺部的免疫炎症反应。低多样性肠道菌群与CIP发生风险增加有关，肠道微生物群落的组成和功能变化可能影响着免疫治疗的效果。Lin等^[56]^通过对165例接受抗PD-1/PD-L1治疗患者的粪便样本进行宏基因组测序，发现其中一种名为苯乙酰谷氨酰胺的代谢产物会削弱抗PD-1治疗的效果。特定的肠道微生物及其代谢产物可能通过调节宿主的免疫微环境进而影响免疫疗效。通过粪菌移植（fecal microbiota transplantation, FMT）可改变患者的肠型，为调节肠道微生物群落来优化免疫疗效提供理论基础。

肠道微生物菌群作为免疫治疗反应的主要肿瘤外调节因子，通过免疫调节、塑造免疫反应、代谢产物介导的纤维化通路与CIP关系紧密。Hazim等^[57]^通过大规模多队列宏基因组分析，揭示了肠道微生物群在不同癌症患者中对ICIs治疗反应的影响，发现了微生物丰度在多种癌症反应者和非反应者中差异显著，并通过体内外实验验证了其免疫调节作用。将对ICIs治疗有反应者的粪便进行FMT能够挽救部分转移性黑色素瘤患者对 ICIs治疗的失败。靶向肠道微生物群可能是改善免疫治疗反应的一种潜在策略。

### 6.3 风险预测模型

近年来机器学习模型在CIP风险分层和早期诊断领域取得了显著突破。梅奥诊所开展了一项跨越8年（2014-2022年）的大规模回顾性队列研究^[58]^，通过整合外周血淋巴细胞绝对值计数（absolute peripheral blood lymphocyte count, ALC）、氧依赖状态、肺功能指标：FVC、FEV_1_、DLCO/pre%及肿瘤PD-L1表达水平等关键变量，采用梯度提升决策树（XGBoost）算法构建全因死亡率的预测模型曲线下面积达0.87（95%CI: 0.72-1.00, P<0.0013）。另外Cousin团队^[50]^能通过整合CT影像组学特征与临床参数，构建广义线性模型和随机森林算法，在预测CIP发生风险方面也展现出中等至良好的性能。

## 7 CIP的预警机制

### 7.1 基线风险评估

CIP是肺癌免疫治疗常见且严重的并发症。临床医生应密切监测CIP患者的早期症状变化，尤其是对于合并高龄、既往存在肺部基础疾病史、联合放化疗等风险因素者在ICIs治疗后前3个月内的典型症状表现。

### 7.2 生物标志物监测与筛选

#### 7.2.1 血清标志物

KL-6作为间质性肺炎的经典早期生物标志物，尤其是在非NSCLC中是CIP的一种可行的生物标志物^[59]^。其他候选标志物如CXCL13、TNF-α、IFN-γ等细胞因子在单细胞测序中被发现显著上调，提示其可能作为动态监测指标^[27]^。细胞因子谱和细胞亚群功能异常亦可能通过诱导肺泡上皮细胞衰老参与肺癌免疫治疗所致的纤维化进展。

#### 7.2.2 自身抗体与T细胞反应

肺泡表面蛋白（surfactant associated protein, SP）（如SP-A、SP-B、SP-D）的自身抗体及特异性T细胞克隆扩增被证实与CIP发生有关，可能成为早期预警的免疫学指标。

#### 7.2.3 肺肠微生物群与代谢组学

肺肠微生物群的失调（如机会性病原体富集）及宿主代谢紊乱可能通过调节局部免疫微环境促进CIP发生^[31]^。

### 7.3 影像学预警特征

ICIs治疗基线时的间质性改变也是CIP重要风险因素，PPFE是CIP预后不良的独立预测因素^[60]^，CIP的影像学特征（如肺纹理特征、间质性肺炎模式等）与非免疫性肺炎、放射性肺炎等均存在一定重叠，遂建议在治疗前、中、后定期进行HRCT检查。影像组学在CIP研究中的核心进展在于利用其量化分析能力解决临床免疫治疗痛点。当前研究的主流一方面在于开发整合影像组学特征在内的预测模型来识别临床ICIs治疗的高CIP风险患者^[61]^；另一方面是构建基于CT影像组学的鉴别诊断模型，以区分在影像学表现上存在重叠及混淆的其他类型肺炎。

### 7.4 多模态预警及临床风险分层模型

现有风险预测模型主要包括传统的逻辑回归模型及近年来开发的机器学习模型，无论是在单中心还是多中心数据上开发和验证的模型均多基于单基线数据，虽然简单易行但预测效能存在局限，忽略了多模态数据整合的探索。目前机器学习驱动的CIP风险分层模型已从单一临床因素分析，演进为整合多模态数据整合式风险分层模型的开发，增强模型在应对CIP的高变异性和复杂性、改善风险预测方面显示出突出优势，但目前CIP相关自身抗体或微生物组学的研究尚少，仍缺乏能尽可能覆盖所有潜在生物标志物的大规模临床验证研究^[28]^。

## 8 小结与展望

CIP是ICIs治疗中需高度重视的并发症，及时识别其危险因素、建立有效的预警策略可显著改善临床CIP管理难题。目前针对CIP的特异性标志物挖掘、疾病动态监测及机制深度研究仍是短板，为突破当前CIP管理的瓶颈，未来研究应从弥合基础研究与临床转化之间的鸿沟出发，着力于以下两个核心方向：（1）特异性生物标志物的挖掘和验证，整合临床及不同ICIs药物特异性机制研究以构建多模态预测模型，开发可直接服务于临床的标准化工具以实现对临床高危CIP患者的早期精准识别。（2）积极推动和利用多组学技术、机器学习、人工智能等在CIP无创动态监测与疗效评估中的应用，驱动精准诊疗及不同ICIs的致病机制研究，为未来CIP的综合管理提供证据和探索方向。
